# Using the Theory of Planned Behaviour to Explain Use of Traditional Chinese Medicine among Hong Kong Chinese in Britain

**DOI:** 10.1155/2015/564648

**Published:** 2015-10-04

**Authors:** Tina L. Rochelle, Steven M. Shardlow, Sik Hung Ng

**Affiliations:** ^1^City University of Hong Kong, Kowloon Tong, Hong Kong; ^2^The University of Salford, Salford M5 4WT, UK; ^3^The University of Keele, Staffordshire ST5 5TB, UK; ^4^Renmin University of China, Beijing 100872, China

## Abstract

The UK Chinese are known for their underutilisation of western healthcare services. Reasons for this underutilisation are complex. The Theory of Planned Behaviour (TPB) is a widely used model of social cognition, which in the present study is being applied to traditional Chinese medicine (TCM) utilisation and satisfaction with TCM services. Two hundred and seventy-two UK Chinese aged between 15 and 91 years (M = 46.55; SD = 18.53) enrolled in the study. TCM utilisation was associated with gender, age, cultural attachment, and subjective norms. TCM users were more likely to be female and older and have a strong attachment to Chinese culture, and be influenced by the views of important others. Findings highlight the potential of the TPB in exploring TCM utilisation, whilst also throwing light on other factors influential in the use of TCM and satisfaction with TCM service provision among Chinese in the UK.

## 1. Introduction

The United Kingdom (UK) Chinese community has grown substantially in recent decades. Ethnic Chinese currently constitute around 0.7% of the total UK population and around 7% of the total nonwhite population, with a population of around 451,500 [1]. The UK Chinese may be at different levels of acculturation as a result of the various influxes of migration to the UK [2, 3]. Many migrants may hold on to their cultural beliefs and practices upon arrival in the UK.

The National Health Service (NHS) is the public face of British healthcare and is the main provider of healthcare in the UK. NHS services are largely free at the point of delivery and are paid for by the British taxpayer [4]. Healthcare in the NHS is provided through the western biomedical disease-centred model. Despite the dominance of western medicine (WM) within the NHS, a very limited range of traditional Chinese medicine (TCM) services is available through the NHS. However, these services are generally provided by health professionals trained in WM, rather than TCM practitioners [4]. TCM provides a holistic approach to healthcare where the object is to achieve mind-body balance [5]. TCM has a preventative approach to illness, which is in contrast to the western biomedical model of illness, which focuses on dealing with illness only when symptoms of ill health have presented themselves [5–7]. Illness in TCM is commonly treated with herbal remedies in contrast to WM's use of what the Chinese perceive to be strong medication [8]. The Chinese model of health and illness emphasises not only the importance of balance, but also the significance of food. The use of various dietary prescriptions as a means of self-medication is both common and widespread [5]. Research indicates dual utilisation of TCM and WM among the Chinese, both at home [6] and overseas [9]. Recent research points to this medical pluralism as an explanation for the low use of WM, implying that use of TCM may lower use of WM services [8, 9]. Despite limited availability of TCM services on the NHS, Chinese migrants tend to utilise those TCM services available from private Chinese herbalists commonly based around local Chinatowns, such as in London and Manchester, as well as herbal packages sent from migrants' relatives [8, 9].

The Theory of Planned Behaviour (TPB) is a social cognitive model that predicts behavioural intention as a proxy of actual behaviour [10]. According to the model, the most important predictor of whether people will perform a given behaviour is the behavioural intention concerning the performance of the behaviour. An individual's intention to perform any given behaviour is determined by three global constructs: attitudes (perceptions of the advantages and disadvantages of performing a behaviour), subjective norms (perceptions of the approval of significant others of performing the behaviour), and perceived behavioural control (perceptions about how much control a person feels they have to perform the behaviour). The TPB has been used to examine a number of different behaviours and has been shown to predict a variety of health behaviours, including dietary supplement behaviour [11], physical activity [12], and breast self-examinations among women [13]. Thus, the validity of the TPB is well established. However, use of the TPB to explore determinants of utilisation of different types of healthcare, specifically among migrants, remains unchartered.

Using a quantitative approach, the present study examines predictors of TCM utilisation and satisfaction with TCM services among Hong Kong Chinese migrants in Britain. Specifically, the following was hypothesised:

1. TCM users will have greater attachment to Chinese culture than nonusers. Nonusers will have a greater attachment to British culture.
2. There will be significant differences between users and nonusers of TCM regarding TPB components, particularly with regard to attitude.
3. Stronger Chinese cultural attachment and favourable attitude towards TCM will be predictive of greater satisfaction with utilisation of TCM services in the UK.

## 2. Methodology

### 2.1. Questionnaire

The questionnaire consisted of three sections: cultural attachment, engagement with the healthcare system, and beliefs about healthcare services, as well as a number of items relating to personal characteristics, such as age, marital status, educational attainment, monthly income, and length of residency.

Items measuring cultural attachment were adopted from a previous validated measure [14]. Attachment to British and Chinese culture was measured using 23 items on a 5-point scale. Items included “Follow Chinese/English traditions and festivals” and “Eat Chinese/Western food” (never to always). Respondents were also asked to describe their identification of British/Chinese identity and culture using items such as “I feel the Chinese/British identity in me is…” (weak to strong) and “I would describe myself as a Hong Konger” (strongly disagree to strongly agree).

Measures of TPB constructs were used to measure beliefs about TCM. Each of the TPB measures was based on standard wording recommended for measuring components of the TPB [10]. Attitude towards TCM utilisation was measured using five semantic differentials: “For me to use TCM is… ‘harmful-beneficial,' ‘unpleasant-pleasant,' etc.” Subjective norms were measured using four items, including “People who are important to me think I should use….” Perceived behavioural control was measured by four items, including “How much control do you have in using TCM?” (no control-complete control). All items were measured on a 5-point scale. Cronbach's alphas for the domains ranged from *α* = 0.6 to *α* = 0.88.

The Chinese SF-12 [15] was used to measure health status. The SF-12 yields both a physical health composite scale (PCS) and mental health composite scale (MCS). One item measured current health status on a 5-point scale (very bad-very good). One item measured experience of using TCM in the UK on a 2-point scale (yes-no). Four items measuring engagement with healthcare in the past six months with a general practitioner (GP), traditional Chinese medicine practitioner (TCMP), hospital outpatient consultation, and hospital inpatient stay were adopted from census questions [16]. These items were measured on a 5-point scale (never, once, 2–4 times, 5–7 times, and >8 times). Another item measured frequency of engagement with different types of UK healthcare services or providers on a 5-point scale (always-never). A further 18 items measured satisfaction with UK TCM healthcare on a 5-point scale (strongly disagree-strongly agree) and were adapted from a previously validated scale originally designed to measure satisfaction with social services in the UK [17].

### 2.2. Procedure

The measure was available for respondents in two languages: Traditional Chinese or English (Traditional Chinese and Simplified Chinese are two standard sets of Chinese characters of the contemporary written form of the Chinese language; while Traditional Chinese is used in Taiwan, Hong Kong, and Macau, Simplified Chinese is officially used in Mainland China, Singapore, and Malaysia). All items in the measure were translated into Chinese from English (SHN) and then backtranslated by another member of the research team (TKN) to ensure accuracy. The measure was piloted among 30 British Chinese across the UK prior to the full implementation in order to improve wording and comprehensibility of the measure. Pilot participants were recruited from a Chinese organisation in Manchester, which also served as one of the main recruitment sites for the study. Minor amendments to the Chinese wording of the measure were made following the pilot. Pilot participants were excluded from the main study. The study received ethical approval from The University of Salford Ethical Review Committee (ref.: REP09/025). Participants were recruited via local Chinese organisations in the UK, predominantly in Manchester and London, two large British cities containing a significant concentration (around 10% and one-third, resp.) of the UK Chinese population. The organisations, comprising Chinese community centres, health centres, and advice centres, were invited to be involved in the study by serving as a recruitment site for participants. All organisations were contacted directly by a member of the research team (TLR). Only those service users identified by the organisation as being Hong Kong Chinese were approached to participate in the study. Respondents received a participant information sheet with detailed information about the study before providing informed consent. The questionnaire was administered by a member of the research team (SHN and TLR).

### 2.3. Analysis

Descriptive analysis was initially performed. Independent samples *t*-tests were used to compare mean scores for the TPB components and cultural attachment variables between users and nonusers of TCM. The influence of age, gender, cultural attachment, TPB components, and recent utilisation behaviour on TCM utilisation and satisfaction with UK TCM services among users of TCM in the UK (including services available on the NHS, private TCM services, and self-prescription) was examined using linear regression. All analyses were carried out using IBM SPSS Statistics 20 (Rochester, New York).

## 3. Results

### 3.1. Sample

Two hundred and seventy-two British Chinese migrants took part in the study, and 56% of participants were female. Respondents ranged in age from 15 to 91 years (M = 46.55; SD = 18.53). Length of residence in Britain ranged from 12 months to 58 years (M = 21.35; SD = 15.14). More than half of respondents' highest educational attainment was a bachelor degree or above. More than half of respondents (53%) were married (see Table 1).

### 3.2. Descriptive Statistics

Forty percent of respondents rated their health as acceptable, relative to their age, while a further 36% rated their health as good. Seventy-six percent of respondents reported experience of using WM, and 24% of respondents had no experience of using WM in the UK. Fifty-nine percent of respondents reported consulting a general practitioner (GP) within the previous six months. Twenty-eight percent of respondents reported having a hospital outpatient appointment in the last six months, and 5% had been a hospital inpatient in the previous six months. One quarter (25%) of respondents reported experience of TCM utilisation in the UK, with 14% having consulted a traditional Chinese medicine practitioner (TCMP) in the last six months.

### 3.3. Independent Samples *t*-Test

Table 2 summarises the differences between users and nonusers of TCM based on reported past behaviour on each of the components of the TPB. Hypothesis 1 was concerned with differences between users and nonusers of TCM regarding cultural attachment. Utilizers of UK TCM reported stronger Chinese cultural attachment, supporting Hypothesis 1, and although nonusers reported stronger attachment to British culture than TCM users, this difference was not significant. TCM users held more positive attitudes towards use of TCM and greater perceived behavioural control over TCM utilisation than nonusers, supporting Hypothesis 2.

### 3.4. Predictors of Utilisation of TCM

A four-block hierarchical regression model was conducted to test the sequential effects of gender and age, cultural attachment, TPB components, and recent TCM utilisation behaviour on predictors of TCM utilisation among HK Chinese in the UK (see Table 3). The purpose of the blocking procedure was to examine whether the addition of subsequent cultural attachment variables, TPB components, and recent utilisation behaviour would add predictive power to the preceding demographic variables. Age and gender entered into block one of the model explained 10% of the variance in TCM utilisation. The entry of cultural attachment variables to block two explained 4% of the variance. The addition of TPB components to the third block explained another 4% of the variance. After the entry of recent utilisation to the fourth and final block, the total variance explained by the model as a whole was 25.3%, *F*(1/193) = 8.17, (*p* < 0.001). Recent TCM utilisation explained an additional 7% of the variance after controlling for age, gender, cultural attachment and the TPB components of attitude, subjective norm, and perceived behavioural control, Δ*R* ^2^ = 0.07, Δ*F*(1/193) = 16.73, (*p* < 0.001). In the final model, gender, age, Chinese cultural attachment, subjective norms, and recent TCM utilisation were all statistically significant in their prediction of TCM utilisation. Recent TCM utilisation recorded the highest beta value in the final model (*β* = 0.26, *p* < 0.001).

### 3.5. Predictors of Satisfaction with UK TCM Services

Hypothesis 3 was concerned with predictors of satisfaction with TCM in the UK. A second four-block hierarchical regression model was conducted to test the sequential effects of gender and age, cultural attachment, TPB components, and recent TCM utilisation behaviour on satisfaction with TCM in the UK (see Table 4). The purpose of the blocking procedure was to examine whether the addition of subsequent cultural attachment variables, TPB components, and recent utilisation behaviour would add predictive power to the preceding demographic variables. Age and gender entered into block one of the model explained 7% of the variance in TCM utilisation. The entry of cultural attachment variables to block two explained 24% of the variance. The addition of TPB components to the third block explained 19% of the variance. After the entry of recent utilisation to the final block, the total variance explained by the model as a whole was 54.6%, *F*(1/46) = 6.91, (*p* < 0.001). Recent TCM utilisation explained an additional 3% of the variance after controlling for age, gender, cultural attachment and the TPB components of attitude, subjective norm, and perceived behavioural control, Δ*R* ^2^ = 0.03, Δ*F*(1/46) = 3.74, (*p* < 0.05). In the final model, gender, age, Chinese cultural attachment, subjective norms, and recent TCM utilisation were all statistically significant in their prediction of satisfaction with UK TCM. Hypothesis 4 posited that Chinese cultural attachment and attitude towards TCM would be predictive of greater satisfaction with UK TCM; this was fully supported by the model. Chinese cultural attachment recorded the highest beta value in the final model (*β* = 0.34, *p* < 0.01).

## 4. Discussion

The present study has identified a range of factors that may influence utilisation of TCM by UK Chinese. Greater utilisation of TCM was associated with being female, being older, having a strong attachment to Chinese culture, subjective norms, and recent TCM utilisation. The findings concur to some extent with the theoretical underpinnings of the TPB. Subjective norms were predictive of TCM utilisation among UK Chinese. However, attitude and perceived behavioural control were not. This does not demonstrate lack of support for the TPB in terms of TCM utilisation among Chinese in the UK per se; rather this leads to other explanations in terms of the relative lack of utilisation of TCM by Chinese migrants. One reason why attitudes were not predictive of TCM utilisation could be that, regardless of whether individuals have a favourable or unfavourable attitude towards TCM and its uses, TCM services are not freely and widely available on the NHS. Individuals need to pay for these services. Previous research demonstrates that the utilisation of TCM services in the UK is costly [8], particularly when compared to Hong Kong or China. This may deter some individuals from utilising these services in the UK, regardless of attitude. The significance of subjective norms is to be expected, given that the Chinese are known to be a collectivistic culture, taking into the account that the views and opinions of important others are particularly important [18]. The importance of subjective norms may have a greater influence than individual attitudes as a result of this collectivistic ideology.

Findings indicate that identity, in the form of cultural attachment, is influential in TCM utilisation. Respondents with strong attachment to Chinese culture were more likely to use TCM and express satisfaction with TCM services. This reinforces previous literature identifying the maintenance of strong cultural links by migrants [19]. Females were more likely to use TCM than their male counterparts. To some extent this is not surprising, given that, with use of medicine and health services in general, women are more likely to use services than men [20]. More so, perhaps with TCM, in that herbal medicine needs to be prepared, the herbs need to be boiled. Thus, it could be argued that females may be more likely to take on the role of preparing TCM, than males, which may go some way to explain the association between higher rates of TCM utilisation among females. Another explanation could be that access to mainstream WM services may be more limited as a result of language barriers. Studies have shown that, despite the provision and availability of translators for medical appointments, some British Chinese, particularly women, do not like to use a translator, feeling uncomfortable with the idea of telling a stranger personal health information [8]. As a result, women may avoid using WM in some cases.

Only a small proportion of respondents reported using TCM exclusively. One reason for this could be that WM is the major face of healthcare provision in the UK. A very small number of TCM services are provided by the NHS, which are generally accompanied by long waiting lists; all other TCM services are run in private practice and thus involve costs which are not applicable if one chooses to use WM. Previous research has pointed to the pragmatic nature of Chinese migrants when it comes to health service utilisation. While migrants may demonstrate more support for the TCM approach, they may use WM regardless of strong beliefs and attitudes towards TCM as WM is available free of charge on the NHS. This may explain the low levels of TCM utilisation among the UK Chinese. Perhaps if TCM was regulated in the same way as WM in the UK and was freely available on the NHS, more migrants would utilise UK TCM services.

The present findings carry practice implications to physicians and professionals working within the NHS. Despite the fact that most participants reported using WM, a quarter of respondents reported using TCM. It is likely that WM and TCM are used simultaneously, as has been previously reported [8, 9]. Health professionals need to take this into account, bearing in mind that a common use of TCM is with herbal remedies. Health professionals should also take into account cultural factors when providing treatment and services. The common practice of utilising both TCM and WM should remind policy makers to consider the regulation of TCM to protect service users.

Limitations of the present study must be acknowledged. The utilisation of convenience sampling, such as the one used in the present study, means that the findings do not readily allow for a generalisation of effects. Whether differences identified in the present patterns of medicinal utilisation would be found in a representative sample of British Chinese migrants remains to be tested. The present study used a convenience sample of British Chinese individuals recruited via British Chinese organisations in large cities in the UK, like Manchester and London. It could be argued that these individuals may be more predisposed to utilise TCM, as evidenced by their strong attachment to Chinese culture by virtue of their association with British Chinese organisations. However, this proved not to be the case in the present study with findings demonstrating relatively low utilisation of UK TCM services.

## Figures and Tables

**Table 1:** Sociodemographic details of respondents.

	Percent
Gender	
Male	44%
Female	56%
Age	
≤15 yrs	0.4%
16–24 yrs	14%
25–44 yrs	30%
45–64 yrs	40%
65–74 yrs	6%
≥75 yrs	8.6%
Marital status	
Single	34%
Married	53%
Separated/divorced	6%
Widowed	7%
Educational attainment	
<primary	14%
GCSE^*∗*^	17%
College	16%
≥bachelor's degree	53%
Monthly income	
<£500^*∗*^	20%
£501–£1,000	15%
£1,001–£2,000	26%
£2,001–£3,000	17%
>£3,000	22%
Length of HK residency (prior to living in UK)	
<10 yrs	7%
10–19 yrs	37%
20–29 yrs	34%
30–39 yrs	13%
40–49 yrs	7%
>50 yrs	2%
Length of UK residency	
<10 yrs	26%
10–19 yrs	26%
20–29 yrs	12%
30–39 yrs	20%
40–49 yrs	12%
>50 yrs	4%

^*∗*^Note: GCSE: General Certificate of Secondary Education; £: British Pound Sterling.

**Table 2:** Comparison between users and nonusers of TCM in the UK [mean (SD)].

	Users (*n* = 65)	Nonusers (*n* = 196)	Significance of difference	95% CI for difference
Chinese cultural attachment^a^	4.35 (0.39)	4.07 (0.61)	<0.001^*∗∗∗*^	0.15 to 0.41
British cultural attachment^a^	3.54 (0.89)	3.57 (0.84)	0.875	−0.26 to 0.22
Attitudes^a^	3.83 (0.78)	3.50 (0.82)	<0.01^*∗∗*^	0.10 to 0.58
Subjective norms	0.51 (0.47)	0.43 (0.46)	0.23	−0.05 to 0.22
Perceived behavioural control^a^	2.75 (0.61)	2.51 (0.50)	<0.01^*∗∗*^	0.08 to 0.39
Self-reported health^a^	3.71 (0.81)	3.59 (0.88)	0.32	−0.12 to 0.37

^*∗∗*^ *p* < 0.01; ^*∗∗∗*^ *p* < 0.001. ^a^Scores are from 1 to 5. Higher scores indicate stronger attachment and so forth.

**Table 3:** Predictors of TCM utilisation among Chinese in the UK.

Variables	Step 1 *β*	Step 2 *β*	Step 3 *β*	Step 4 *β*
Female	0.21^*∗∗*^	0.23^*∗∗∗*^	0.22^*∗∗*^	0.22^*∗∗∗*^
Age	0.20^*∗∗*^	0.20^*∗∗*^	0.22^*∗∗*^	0.20^*∗∗*^
Chinese cultural attachment		0.19^*∗∗*^	0.14^*∗*^	0.16^*∗*^
British cultural attachment		0.10	0.10	0.10
Attitudes			0.01	−0.04
Subjective norms			0.14^*∗*^	0.14^*∗*^
Perceived behavioural control			0.13	0.12
Recent utilisation behaviour				0.26^*∗∗∗*^

Δ*R* ^2^	0.10	0.04	0.04	0.07
df.	2/199	2/197	3/194	1/193
Δ*F*	10.45^*∗∗∗*^	4.95^*∗∗*^	3.36^*∗*^	16.73^*∗∗∗*^

^*∗*^ *p* < 0.05; ^*∗∗*^ *p* < 0.01; ^*∗∗∗*^ *p* < 0.001.

**Table 4:** Predictors of satisfaction with UK TCM services among Chinese in the UK.

Variables	Step 1 *β*	Step 2 *β*	Step 3 *β*	Step 4 *β*
Female	0.27^*∗*^	0.32^*∗∗*^	0.25^*∗*^	0.25^*∗*^
Age	−0.04	−0.10	−0.05	−0.07
Chinese cultural attachment		0.49^*∗∗∗*^	0.33^*∗∗*^	0.34^*∗∗*^
British cultural attachment		0.10	0.10	0.09
Attitudes			0.28^*∗*^	0.24^*∗*^
Subjective norms			0.20	0.20
Perceived behavioural control			0.18	0.17
Recent utilisation behaviour				0.20^*∗*^

Δ*R* ^2^	0.07	0.24	0.19	0.03
df.	2/52	2/50	3/47	1/46
Δ*F*	2.00	8.92^*∗∗∗*^	6.17^*∗∗∗*^	3.74^*∗*^

^*∗*^ *p* < 0.05; ^*∗∗*^ *p* < 0.01; ^*∗∗∗*^ *p* < 0.001.
